# Epidemiology of Pathogenic Enterobacteria in Humans, Livestock, and Peridomestic Rodents in Rural Madagascar

**DOI:** 10.1371/journal.pone.0101456

**Published:** 2014-07-01

**Authors:** DeAnna C. Bublitz, Patricia C. Wright, Jonathan R. Bodager, Fidisoa T. Rasambainarivo, James B. Bliska, Thomas R. Gillespie

**Affiliations:** 1 Centre ValBio, Ranomafana, Madagascar; 2 Department of Molecular Genetics and Microbiology, Center for Infectious Diseases, Stony Brook University, Stony Brook, New York, United States of America; 3 Department of Anthropology, Stony Brook University, Stony Brook, New York, United States of America; 4 Department of Environmental Health, Rollins School of Public Health, Emory University, Atlanta, Georgia, United States of America; 5 Department of Environmental Sciences and Program in Population Biology, Ecology, and Evolution, Emory University, Atlanta, Georgia, United States of America; University of Minnesota, United States of America

## Abstract

**Background:**

Among the families of enteric bacteria are globally important diarrheal agents. Despite their potential for zoonotic and environmental transmission, few studies have examined the epidemiology of these pathogens in rural systems characterized by extensive overlap among humans, domesticated and peridomestic animals. We investigated patterns of infection with Enterotoxigenic *Escherichia coli*, *Shigella* spp., *Salmonella enterica*, *Vibrio cholerae*, and *Yersinia* spp. (*enterocolitica*, and *pseudotuberculosis*) in Southeastern Madagascar where the potential for the aforementioned interactions is high. In this pilot project we conducted surveys to examine behaviors potentially associated with risk of infection and if infection with specific enterobacteria species was associated with diarrheal disease.

**Methodology/Principal Findings:**

PCR was conducted on DNA from human, livestock, and rodent fecal samples from three villages. Overall, human prevalence was highest (77%), followed by rodents (51%) and livestock (18%). Rodents were ∼2.8 times more likely than livestock to carry one of the bacteria. The incidence of individual species varied between villages, with the observation that, *E. coli* and *Shigella* spp. were consistently associated with co-infections. As an aggregate, there was a significant risk of infection linked to a water source in one village. Individually, different pathogens were associated with certain behaviors, including: those who had used medication, experienced diarrhea in the past four weeks, or do not use toilets.

**Conclusions/Significance:**

Different bacteria were associated with an elevated risk of infection for various human activities or characteristics. Certain bacteria may also predispose people to co-infections. These data suggest that a high potential for transmission among these groups, either directly or via contaminated water sources. As these bacteria were most prevalent in humans, it is possible that they are maintained in humans and that transmission to other species is infrequent. Further studies are needed to understand bacterial persistence, transmission dynamics, and associated consequences in this and similar systems.

## Introduction

Enteric diseases are a leading cause of illness and death in the developing world. Gastric infections and diarrhea are estimated to account for 2.2 million global mortalities annually. Diarrheal pathogens present an exceptional threat to children under 5, for whom nearly 15% of all deaths can be attributed to diarrhea, making it the second leading cause of death for infants worldwide [1], [2]. Additionally, disease caused by these infections results in years of life lost due to malnutrition and stunted growth, both physical and cognitive [2]. These infections are especially prevalent in the developing world, particularly Africa, where infection-related diarrhea accounts for as much as 8.5% of all fatalities [3]. A recent 3-year study evaluating over 9000 children with moderate to severe diarrhea in Africa and Asia found that Enterotoxigenic *E. coli* and *Shigella* were two of the four most common causes of infection. *V. cholerae* was also identified as a significant cause of diarrhea in certain sites [4].

The source of these pathogens can be varied, though the interplay among humans, companion and food animals, and peridomestic animals is increasingly being recognized as a key interface for disease transmission [5]–[7]. As human populations grow they are increasingly pressed into higher density living. The same is true for the agriculture and livestock needed to support these communities. Moreover, in poorer communities there is a greater incidence of peridomestic rodents living in and around homes and food sources [8], [9]. Residing in closer quarters creates a greater chance for the transmission of infectious diseases between humans, livestock, and rodents [5]. Numerous epidemics and pandemics have been linked to the cycle of transmission from livestock and humans including avian flu, Nipah virus, and swine flu [10]–[12]. Rodents present a similar risk as they can be the source of a wide range of diseases including tularemia, *Cryptosporidium* spp., *Campylobacter jejuni*, and hantavirus [7]. Furthermore, rodents are host to parasites such as ticks and fleas that can perpetuate the cycle of other pathogens like Lyme disease and plague [13], [14]. Despite the high potential for zoonotic transmission, these interactions among humans, livestock, and peridomestic animals are still relatively understudied.

Madagascar is a nation of ∼22 million people. While efforts have been made to improve living conditions for the people of Madagascar, only ∼50% of the population is using improved water sources and less than 20% have access to improved sanitation facilities [15]. Diarrheal diseases cause approximately 37% of all infection and parasite-related deaths each year in this country [16]. A recent study investigating diarrheal disease in Madagascar found that nearly 50% of children under 5 tested positive for pathogenic intestinal microorganisms and roughly 10% of this age group died from diarrhea-related illness [15], [17].

Much of the Malagasy population is rural and relies on livestock and rice farming for subsistence. Anthropogenic disturbance associated with agricultural practices and timber harvesting has led to nearly 90% of Madagascar's forest being lost [18], [19]. Habitat loss and fragmentation can be devastating to biodiversity and endemic species but creates ideal spaces for invasive and generalist species such as introduced rodents [20]–[23]. Disruption of the natural environment coupled with high-density living presents an increased chance for contact with rodents and thus, the potential for disease transmission to both humans and livestock. Hantavirus, which can be transmitted as an aerosol from rodent urine, becomes more prevalent with habitat disturbance [7], [21], [22]. In regions where sanitation infrastructure is lacking, these interactions are all the more likely, exacerbating one's risk of infection with a zoonotic or waterborne pathogen [1], [24]. In addition, zoonotic *Yersinia pestis*, the causative agent of plague, is endemic to Madagascar. It is estimated that 40% of the population is exposed to *Y. pestis*, which can be carried by rats and transmitted via aerosol or flea bites, resulting in severe and often fatal illness [25], [26]. However, as this is not typically an enteric pathogen it is not covered in this study.

This pilot project was undertaken to look at the prevalence of five pathogenic enterobacteria: Enterotoxigenic *Escherichia coli* (ETEC), *Shigella* spp. (*flexneri* and *dysenteriae*), *Salmonella enterica*, *Vibrio cholerae*, and *Yersinia* spp. (*enterocolitica*, and *pseudotuberculosis*) in humans, livestock, and rodents. These groups were sampled from three villages near Ranomafana National Park: Ambatolahy (Amb), Ambodiaviavy (Avy), and Ankialo (Ank), in Southeastern Madagascar. The work herein estimates prevalence levels for these five bacteria in the above mentioned sample groups and looks at the relative risk association to various activities to identify potential sources or patterns of transmission. As humans interact with, and alter, their environment it becomes increasingly important to understand the risks in regards to pathogen transmission to and from all potential hosts and their environment. Our results highlight a little studied facet of disease ecology in Madagascar and suggest a relationship between humans, livestock, and rodents in propagating zoonotic and waterborne pathogens.

## Methods

### Ethics Statement

All protocols, including obtaining oral consent from participants, were reviewed and approved by the Ministry of Health of the government of Madagascar, the Stony Brook University Internal Review Board and Institutional Animal Care and Use Committee. As approved by the Stony Brook Internal Review Board, oral informed consent of participants was obtained prior to specimen collection and survey. In the case of minors, a parent or guardian provided informed consent. Given the low literacy rate of the population being studied, we opted for oral consent administered and recorded on the survey sheets by the native interpreter conducting the interview. All participants were anonymously given unique identifiers. Permits were not required for sample collection from the animals in this study. All cows and pigs sampled were handled according to the guidelines of the National Veterinary Services Laboratories (Publication N231597), USDA, Fort Collins, Colorado. Rodents were handled following protocols outlined by the CDC [27]. No endangered species were involved in this study.

### Study Site

The study took place in and around Ranomafana National Park, Madagascar, (located 47°18′ 40 to 47°37′E and 21°2′ to 21°25′S) a 43,500 hectare World Heritage Site well known for its high levels of species endemism and diversity [28], [29]. Three communities located on the edge of the park were selected as the focus of this study: Ambodiaviavy (Avy, population  = 363), Ankialo (Ank, population  = 361), and Ambatolahy (Amb, population  = 256). The communities are located in different areas of the park and have distinct cultural practices. The study population included several peridomestic rodents (*Rattus rattus, Mus musculus*), bovine (*Bos indicus*), porcine (*Sus domesticus*), and humans.

### Sample collection and surveys

In June and July 2011, household and individual surveys were administered in the three communities: Avy (n = 65, total households = 10), Ank (n = 70, total households = 10), and Amb (n = 47, households = 10). A cluster sampling method was used: in each village, ten households were selected and every person inhabiting a selected household was surveyed. Participants were chosen independent of age, sex, or symptoms, but some were selected based on livestock ownership. Surveys were comprehensive with inquiries of demographic information, health status, hygiene, medication usage, water usage, and exposure to livestock and wildlife (70 questions for individual survey and 40 variables for the household survey). Potential behaviors associated with risk for diarrheal disease and infection with enterobacteria were queried in both surveys. Trained local field assistants administered all surveys in the local language (Malagasy) in order to reduce survey bias. Data were recorded on paper forms, answers were converted from “Yes/Always,” “Sometimes,” or “No/Never,” to a 2, 1, or 0, respectively. These numbers were entered into Microsoft Excel spreadsheets with the associated behavior, and reviewed for accuracy. Fisher's exact test using Prism 6 (Graphpad, La Jolla, CA) was used to analyze associations and calculate confidence intervals (CI) and relative risk (RR) between survey responses and infection status.

All survey participants were asked to provide a fecal specimen for examination of diarrheal pathogens and 89% complied. Concurrently, domesticated animals of participants (bovine and porcine) were sampled and baited rodent live-traps were set inside participant homes overnight. The following morning, fecal specimens were collected from trapped peridomestic rodents. All of the traps were washed thoroughly with 10% bleach solution between uses. Human volunteers were instructed to wash their hands prior to collecting fecal samples in sealable plastic bags that were collected the same or the next day. Given that we did not have access to a clinic and were limited in time at each village, we cannot assure 100% sterile transfer of the samples from the participants into the bags. Fresh fecal matter from livestock was collected from the rectum using a non-sterile latex glove, or from the ground if defecation was observed. For the latter, only fecal material that had not directly been in contact with the ground was collected. All samples were moved to a field laboratory as soon as possible (Amb and Avy) or processed on site (Ank). Approximately one milliliter of feces from each sample was homogenized with an equal volume of RNAlater nucleic acid stabilizing buffer (Ambion, Life Technologies, Grand Island, NY) and stored at −20°C at CentreValBio until transport to the United States.

### Molecular methods

Total nucleic acid was extracted from fecal specimens (n = 278) preserved in RNAlater using the FastDNA SPIN Kit for Soil (MP Biomedicals, LLC, Solon, OH), following the manufacturer-recommended procedures. Using PCR, we screened the samples for ETEC, *S. enterica*, *Shigella* spp. (*flexneri* and *dysenteriae*), *V. cholerae*, and *Yersinia* spp. We chose to amplify the gene *yadA* in *Yersinia* as it is similar in *enterocolitica*, and *pseduotuberculosis* species and could be used to screen for both simultaneously [30]. The *yadA* primers should generate a product of 849 bp with *Y. enterocolitica* serogroup 03/09 strains, a product of 759 bp ith *Y. enterocolitica* serogroup 08 strains, and 681 bp product with *Y. pseudotuberculosis* (including the positive control strain used). Likewise, the *invA* gene in *S. enterica* was used for its conserved nature across serovars [31]. A portion of the ipaH gene was amplified to detect *Shigella flexneri* and *dysenteriae*. For positive controls in the PCR reactions, *V. cholerae*, ETEC, and *S. flexneri* strains were obtained from American Type Culture Collection (ATCC, Manassas, VA). The 32777 strain in the Bliska Laboratory collection was used as a positive control for *Y. pseudotuberculosis*. The *S. enterica* serovar 14028 positive control strain was obtained from the laboratory of Dr. Adrianus van der Velden (Both: Stony Brook University, Stony Brook, NY). All positive control strains are listed in Table 1. Genomic DNA was isolated from each of the strains using a DNeasy kit (Qiagen, Valencia, CA) according to the manufacturer's protocol. All primers are listed in Table 1 and detect previously described genes for each bacterial species [26], [31], [32]. The primers were synthesized by Eurofilms MWG Operon (Stony Brook University, Stony Brook, NY). PCR was conducted on 2.0 µl of DNA sample using 0.5 µmol of each primer (Table 1) in 25 µl of Platinum PCR mix (Invitrogen, Life Technologies, Grand Island, NY). As a negative control, the same PCR reaction was run using water to confirm that there was no contamination of the reagents. Additionally, all of the primers were tested on all of the positive control strains to test for cross-reactivity and none was found. The amplification setting was as previously described; sensitivity of detection for each pathogen is listed in Table 1 [32].

**Table 1:** Bacterial strains (positive controls), target genes, and primers* used in this study.

Genus and Species (ATCC #)	Target Gene	PCR primers (5′-3′)	Product Size	Sensitivity (cells)
Enterotoxigenic *E. coli* serotype O78:H11 (35401)	Enterotoxin (LT) gene	f - GAGACCGGTATTACAGAAATC r - GAGGTGCATGATGAATCCAG	117 bp	40
*Shigella flexneri* serotype 2b (12022)	*ipah*	f - CTTGACCGCCTTTCCGATAC r - CAGCCACCCTCTGAGAGTA	610 bp	5×10^4^
*Salmonella enterica* serovar Typhimurium (14028)	*invA*	f - TATCGCCACGTTCGGGCAA r - TCGCACCGTCAAAGGAACC	275 bp	40
*Vibrio cholerae* (14035)	*ctxA*	f - GGCAGATTCTAGACCTCCT r - TCGATGATCTTGGAGCATTC	563 bp	40
*Yersinia pseudotuburculosis* serogroup O1 (32777)	*yadA*	f - CTTCAGATACTGGTGTCGCTGT r - ATGCCTGACTAGAGCGATATCC	681 bp (849^a^, 751^b^)	Unknown

*All primer sequences and sensitivities obtained from Wang et al. (1997) except *Yersinia* obtained from Thoerner et al. (2003).

a Product size with *Y. enterocolitica* serogroup 03 or 09 strains.

b Product size with *Y. enterocolitica* serogroup 08 strains.

## Results

For this pilot study a cluster sampling method was used: in each village, ten households were selected and every person inhabiting a selected household was surveyed. Participants were chosen independent of age, sex, or symptoms. Participating households were selected at random except that preference was given to households owning livestock. Overall, 304 fecal samples were tested (humans  = 163, cattle  = 58, pigs  = 18, and rodents  = 65). Of the species sampled, humans had the highest overall prevalence of infection (77%, CI = 0.70–0.83), followed by rodents (51%, CI = 0.38–0.63), then livestock (18%, CI = 0.10–0.29) (Table 2). When looking at the prevalence of each pathogen individually, there was variability among villages. Among humans, *Shigella* spp. was the predominant pathogen detected in Amb (64%, CI = 0.48–0.77), while ETEC was the dominant bacterium found in human samples from Avy and Ank (69%, CI = 0.55–0.80 and 57%, CI = 0.43–0.70, respectively) (Table 3).

**Table 2:** Prevalence of Enterobacteriaceae infection by subject and location.

Host	Location							
	Ambatolahy		Ambodiaviavy		Ankialo		All Villages	
	+/Total	Prevalence	+/Total	Prevalence	+/Total	Prevalence	+/Total	Prevalence
**Human**	43/47	0.91	42/58	0.72	41/58	0.71	126/163	0.77
**Cattle**	0/14	0.00	2/17	0.12	0/27	0	2/58	0.03
**Pig**	4/4	1.00	1/1	1.00	7/13	0.54	12/18	0.67
**Rodent**	12/33	0.36	10/14	0.71	11/18	0.61	33/65	0.51

**Table 3:** Incidence of each Enterobacteriaceae species in humans, livestock, and rodents by village.

	Ambatolahy					
Pathogen	Human		Livestock*		Rodent	
	+/Total	Prevalence	+/Total	Prevalence	+/Total	Prevalence
***E. coli***	20/47	0.43	4/29	0.14	10/33	0.30
***Shigella*** ** spp.**	30/47	0.64	0/29	0.00	4/33	0.12
***S. enterica***	15/47	0.32	0/29	0.00	6/33	0.18
***V. cholerae***	1/47	0.02	0/29	0.00	0/33	0.00
***Yersinia*** ** spp.**	13/47	0.28	0/29	0.00	0/33	0.00

	Ambodiaviavy					
	Human		Livestock*		Rodent	
	+/Total	Prevalence	+/Total	Prevalence	+/Total	Prevalence
***E. coli***	40/58	0.69	2/18	0.11	8/14	0.57
***Shigella*** ** spp.**	32/58	0.55	0/18	0.00	5/14	0.36
***S. enterica***	9/58	0.16	2/18	0.11	6/14	0.43
***V. cholerae***	10/58	0.17	0/18	0.00	2/14	0.14
***Yersinia*** ** spp.**	8/58	0.14	0/18	0.00	0/14	0.00

	Ankialo					
	Human		Livestock*		Rodent	
	+/Total	Prevalence	+/Total	Prevalence	+/Total	Prevalence
***E. coli***	33/58	0.57	6/40	0.15	8/18	0.44
***Shigella*** ** spp.**	15/58	0.26	6/40	0.15	10/18	0.56
***S. enterica***	15/58	0.26	6/40	0.15	5/18	0.28
***V. cholerae***	10/58	0.17	1/40	0.03	0/18	0.00
***Yersinia*** ** spp.**	6/58	0.10	0/40	0.00	0/18	0.00

*Cattle and pigs.

In regards to livestock, ETEC and *S*. *enterica* were the only enterobacteria detected in samples from Amb and Avy, while *Shigella* spp., *V. cholerae*, as well as the previous two, were found in samples from Ank (Table 3). These pathogens were more prevalent in pigs than cattle (67%, CI = 0.41–0.87 and 3%, CI = 0.004–0.12, respectively). Overall, few livestock tested positive for any of the target pathogens (Amb = 14%, Avy = 11%, Ank = 15%).

Except for *Yersinia*, all target species were detected in rodent samples, though *V. cholerae* was only found in rodent samples from Avy (Table 3). The occurrence of infection in rodents was 36% (CI = 0.20–0.55) in Amb, 71% (CI = 0.42–0.92) in Avy, and 61% (CI = 0.36–0.83) in Ank (Table 2). However, it should be noted that fewer rodents were sampled in either Avy or Ank versus Amb (n = 14, 18, and 33, respectively).

As depicted in Table 2, prevalence of enterobacteria infection was markedly higher for residents of Amb. This was linked to an increased risk association of having one or more of the pathogens for the residents of Amb vs. the residents of Avy or Ank (RR = 1.279, p = 0.0066) (Table 4). However, when broken down by pathogen, there was a reduced risk of *V. cholerae* and ETEC infection in Amb (*V. cholerae* RR = 0.0840, p = 0.0003; *E. coli* RR = 0.6762, p = 0.0229). Conversely, there was a higher risk of infection with these two pathogens in Avy (*V. cholerae* RR = 3.26, p = 0.0003; ETEC RR = 1.366, p = 0.0313). There was an elevated chance of *Shigella* spp.infection in Amb (RR = 1.575, p = 0.0092) compared to the other two villages, with a dramatically reduced risk in Ank (RR = 0.438, p<0.0001). Lastly, there was a higher prevalence of *Yersinia* spp. in humans from Amb versus Avy or Ank (RR = 2.241, p = 0.0344)(Table 4). All but one sample yielded a product of 681 bp in size indicating that *Y. pseudotuberculosis* is likely the species present. The one outlier was 759 bp suggesting infection with *Y. enterocolitica*.

**Table 4:** Risk factors for infection with Enterobacteriaceae in people living in villages in Southeast Madagascar.

			95% CI		
Variable	n*	RR	lower	upper	p
Age (≤15)	162	1.079	0.898	1.297	0.486
Sex (male vs. female)	162	1.036	0.875	1.227	0.7103
**Amb vs. Ank or Avy**	**163**	**1.279**	**1.107**	**1.477**	**0.0066**
Avy vs. Amb or Ank	163	0.9052	0.7519	1.09	0.3292
Ank vs. Amb or Avy	163	0.8732	0.7221	1.056	0.1715
**Amb vs. Ank or Avy ** ***V. cholerae***	**163**	**0.0841**	**0.0118**	**0.5988**	**0.0003**
**Amb vs. Ank or Avy ** ***E. coli***	**163**	**0.6762**	**0.4716**	**0.9696**	**0.0229**
**Amb vs. Ank or Avy ** ***Shigella*** ** spp.**	**163**	**1.575**	**1.158**	**2.144**	**0.0092**
**Amb vs. Ank or Avy ** ***Yersinia*** ** spp.**	**163**	**2.241**	**1.144**	**4.389**	**0.0344**
**Avy vs. Amb or Ank ** ***V. cholerae***	**163**	**3.26**	**1.682**	**6.321**	**0.0003**
**Avy vs. Amb or Ank ** ***E. coli***	**163**	**1.366**	**1.057**	**1.766**	**0.0313**
**Ank vs. Amb or Avy ** ***Shigella*** ** spp.**	**163**	**0.438**	**0.2754**	**0.6966**	**<0.0001**
Collects water from an open source (vs. closed well or pump)	119	0.8503	0.6876	1.052	0.1387
**Avy only - collects water from open source**	**38**	**2.174**	**1.146**	**4.122**	**0.0041**
Boils water	163	0.9678	0.8197	1.143	0.7126
Washes hands prior to eating	163	0.9385	0.7664	1.149	0.8016
Uses a toilet	163	1.119	0.9441	1.327	0.449
Works in agricultural fields	153	0.9744	0.7503	1.266	1
Tends livestock	144	0.9198	0.7601	1.113	0.4386
Contact with rodents	151	1.083	0.911	1.288	0.4369
Experienced diarrhea (vs. no diarrhea) in past 4 Weeks	159	1.114	0.9155	1.357	0.4453
Experienced diarrhea with blood (vs. no blood) in past 4 weeks	25	0.7719	0.3409	1.748	0.4217
Used medicine (traditional or commercial) in past 4 Weeks	163	1.064	0.8684	1.303	0.5279
**Rodents vs. livestock**	**rodent-65 livestock-76**	**2.756**	**1.622**	**4.684**	**<0.0001**

*Total n varies due to incomplete notation on some surveys or respondents do not participate in the given activity (e.g. tend livestock).

**Bold**  =  statistically significant associations.

Given that 77% of humans tested were found to be carrying at least one of these pathogens, we wanted to examine the prevalence of co-infections in this population. In Amb, none of the bacteria were significantly linked to one another while samples from Avy and Ank both showed patterns of co-infection (Table 5). In Avy, 86% of ETEC and 97% of *Shigella* spp. positive samples were also positive for one of the other bacteria tested, of which ETEC co-infection with *Shigella* spp was the most common (Prevalence  = 75%, p<0.0001) (Table 5). While in Ank, *S. enterica* and *Shigella* spp. were both equally associated with co-infection with 100% of those infected with either pathogen testing positive for at least one other enterobacteria (p = 0.0029) and 60% of those were co-infections of *Shigella* spp. and *S. enterica* (p = 0.0011) (Table 5). *Shigella* spp. was also significantly linked to infection with *Yersinia* spp. in Ank (p = 0.0338). Overall, co-infections involving ETEC or *Shigella* spp. were most common at a prevalence of 74% and 91% respectively, across all three villages. ETEC co-infections with *Shigella*/EIEC was the most common with 55% of total ETEC infections co-occurring with *Shigella* spp. (p = 0.0276) (Table 5).

**Table 5:** Prevalence of Enterobacteriaceae co-infections in humans from villages in Southeastern Madagascar.

	Ambodiaviavy			
	*E. coli*		*Shigella* spp.	
	Prevalence	p	Prevalence	p
***Shigella*** ** spp.**	0.75	<0.0001	N/A	N/A
***S. enterica***	0.23	0.0454	0.25	0.0333
***V. cholerae***	0.48	0.0022	0.56	<0.0001
***Yersinia*** ** spp.**	0.20	0.0484	0.25	0.0063
**All enterics**	0.86	<0.0001	0.97	<0.0001

	Ankialo			
	*Shigella* spp.		*S. enterica*	
	Prevalence	p	Prevalence	p
***Shigella*** ** spp.**	N/A	N/A	0.60	0.0011
***S. enterica***	0.60	0.0011	N/A	N/A
***Yersinia*** ** spp.**	0.27	0.0338	N/A	N/A
**All enterics**	1.00	0.0029	1.00	0.0029

	All Villages			
	*E. coli*		*Shigella* spp.	
	Prevalence	p	Prevalence	p
***Shigella*** ** spp.**	0.55	0.0276	N/A	N/A
***S. enterica***	0.30	0.0413	0.38	0.0001
***V. cholerae***	0.26	0.0148	0.31	0.0002
***Yersinia*** ** spp.**	N/A	N/A	0.25	0.0109
**All enterics**	0.74	0.0010	0.91	<0.0001

Broadly, none of the potential risk factors that we analyzed, including age, sex, working in fields, washing hands or boiling water, were significantly associated with an increased risk of infection in humans (Table 4). While there was no overall risk tied to fetching water from an open vs. a closed source in the three villages combined (RR = 0.8503, p = 0.1387), there was a substantial increase in risk of infection linked to collecting water from a closed source in Avy (RR = 2.174, p = 0.0041). Additionally, infection with ETEC was significantly linked to fetching water from a closed source (RR = 1.977, p = 0.0087). However, other activities, such as having experienced diarrhea within the four weeks prior to the survey, or tending livestock were not connected to an amplified risk of infection when all pathogens are considered as an aggregate (Table 4).

Certain factors and activities were associated with an elevated risk of infection when the pathogens are evaluated individually. There was an increased risk of *Shigella* spp. for individuals 15 years of age or under in Amb (RR = 1.655, p = 0.0355) while this group carried a greater risk of infection by ETEC in Avy (RR = 1.450, p = 0.0487) (Table 6). In Avy there was also a modest association between infection with ETEC and individuals who did not boil their water (RR = 1.447, p = 0.0487) as well as those who had used medication (RR = 1.826, p = 0.0348) or experienced diarrhea (RR = 1.513, p = 0.0438) in the past four weeks. Of those who had used medication, over 76% had used antibiotics (anti-bacterial, -protozoal, and – helmintic), 78% had used anti-inflammatories, and 52% had used both. There was a 3.8 and 4.5 times greater risk of infection with *V. cholerae* in Amb and Ank, respectively, for individuals who reported never using a toilet (Amb p = 0.022; Ank p = 0.0318). Lastly, when all three villages were combined, males were 2.14 times more likely to be infected with *S. enterica* (p-0.016). Additionally, people who never used a toilet carried a greater risk of infection with *Yersinia* spp. (RR = 3.575, p = 0.0013). Importantly, having suffered from diarrhea in the past four weeks was significantly associated with infection with *V. cholerae* (RR = 2.622, p = 0.0156) (Table 6).

**Table 6:** Risk factors for infection with individual Enterobacteriaceae species in people living in villages in Southeast Madagascar.

Ambatolahy						
				95% CI		
Variable	Pathogen	n*	RR	lower	upper	p
Age (≤15)	*Shigella* spp.	47	1.655	1.031	2.658	0.0355
Does not use a toilet	*Yersinia* spp.	47	3.818	1.019	14.30	0.022

Ambodiaviavy						
				95% CI		
Variable	Pathogen	n*	RR	lower	upper	p
Age (≤15)	*E. coli*	58	1.45	1.014	2.073	0.0487
Collects water from an closed source (pump, well)	*E. coli*	58	1.977	1.044	3.744	0.0087
Used medicine (traditional or commercial) in past 4 Weeks	*E. coli*	58	1.826	0.9169	3.637	0.0348
Does not boil water	*E. coli*	57	1.447	1.027	2.04	0.0478
Experienced diarrhea (vs. no diarrhea) in past 4 Weeks	*E. coli*	58	1.513	1.148	1.994	0.0438

Ankialo						
				95% CI		
Variable	Pathogen	n*	RR	lower	upper	p
Does not use a toilet	*V. cholerae*	58	4.543	1.680	12.28	0.0318

All Villages						
				95% CI		
Variable	Pathogen	n*	RR	lower	upper	p
Sex (male vs. female)	*S. enterica*	162	2.14	1.145	4.000	0.016
Uses a toilet	*Yersinia* spp.	163	3.575	1.868	6.843	0.0013
Experienced diarrhea (vs. no diarrhea) in past 4 Weeks	*V. cholerae*	163	2.622	1.393	4.934	0.0156

*Total n varies due to incomplete notation on some surveys or respondents do not participate in the given activity.

Rodents tested had a greater risk of carrying one of these bacteria compared to livestock. With roughly equal numbers of samples, the RR of a rodent testing positive for one of the infectious agents was 2.756 times greater than that of the livestock tested (RR  = .3628, p<0.0001). There was also a modest positive association between rodents in Amb and carrying at least one of the five pathogens as compared to rodents in Avy or Ank (RR  = 0.5541, p = 0.0259). Additionally, there was no associated risk for humans having touched rodents by their tail (RR = 1.083, p = 0.4369) (Table 4). All of the significant findings have been summarized in Table 7.

**Table 7:** Summary of significant findings for infection with Enterobacteriaceae in human, livestock, and rodent populations.

Ambatolahy
Increased risk of *E. coli*, *Shigella* spp., *V. cholerae*, and *Yersinia* spp.
Increased risk for people 15 years or younger
Increased risk for people who did not use a toilet

Ambodiaviavy
Increased risk of *E. coli* and *V. cholerae*
Increased risk for people collecting water from an open source
Increased risk for people 15 years or younger
Increased risk for people who used medicine in past four weeks
Increased risk for people who did no boil their water
Increased risk for people who experienced diarrhea in past four weeks
Co-infection association between *E. coli* and *Shigella* spp.

Ankialo
Increased risk of *Shigella* spp.
Increased risk for people who did not use a toilet
Co-infection association between *S. enterica* and *Shigella* spp.
Co-infection association between *Shigella* spp. and *Yersinia* spp.

Additional risk factors (across all villages)
Higher prevalence of bacteria in rodents vs. livestock
Higher prevalence of bacteria in pigs vs. cattle
Males at higher risk for *S. enterica* than females
People who used a toilet at higher risk for *Yersinia* spp.
People who experienced diarrhea in past four weeks at higher risk for *V. cholerae*

## Discussion

This study evaluated the prevalence of five pathogenic bacteria in humans, livestock, and rodents from three villages in Madagascar. Of the three sample groups, humans carried the highest prevalence (77%) followed by rodents (51%), and livestock (18%). The incidence of each pathogen varied from village to village with people from Amb carrying the greatest RR of infection (Table 4). Additionally, the distribution of the bacteria was different in livestock versus rodents with rodents testing positive for more of the pathogens and having a greater overall RR than livestock in all three villages (Table 4).

For many parts of the world, such as Madagascar, enteric pathogens are a major source of illness and death [1], [2], [15], [17]. The impact of humans on their environment and the implications of those alterations on disease transmission are becoming more and more clear. Madagascar is an island with incredible species diversity across all taxa, of which a majority are endemic [33], [34]. This country has also seen drastic changes to the original environment for agriculture and resource extraction, leading to fragmenting or clearing nearly 90% of the original forestland [18], [19], [34]. Overall, zoonotic pathogens account for nearly 61% of the organisms infectious to humans and 75% of emerging pathogens in the last decade [35]. While populations of humans, livestock, and rodents have been living together for thousands of years, these alterations to the landscape and ecology of Madagascar are an opportunity for new interactions between these populations, and potentially the transmission of zoonoses or waterborne pathogens.

This pilot study focused on the villages of Ambatolahy, Ambodiaviavy, and Ankialo near Ranomafana National Park in Southeastern Madagascar. Our results demonstrated that humans accounted for the greatest number of positive samples as compared to livestock or rodents (Table 2). Of the five pathogens tested, ETEC was the most prevalent in humans and livestock. When broken down by village, ETEC was also the dominant pathogen detected in human samples from Avy and Ank, while *Shigella* spp. was the predominant bacterium found in Amb (Table 3). It should be noted that Enteroinvasive *E. coli* (EIEC) strains can also have and express the toxin encoded by the ipaH gene. Having used the ipaH gene to screen for *Shigella* spp. it is also possible that the bacteria detected were EIEC. However, the disease caused by EIEC is nearly identical to Shigellosis and still presents an equal threat to the health of these communities [36]. This would be an important avenue to follow up on with sequencing of the samples to establish which species are present in these populations.

Globally, information about multiple enterobacteria infections is lacking, as such, this study also examined the prevalence of co-infections in humans. ETEC and *Shigella* spp. were predominantly associated with co-infections with 74% of ETEC and 91% of *Shigella* spp. infected samples testing positive for at least one other enterobacteria species (Table 5). These same pathogens were also the most prevalent individually in Amb and Avy while in Ank, *S. enterica* and *Shigella* spp. were present in equal amounts and both linked to co-infection in that village (Table 3, 5). Given that the numbers were identical for *S. enterica* and *Shigella* spp.in Ank, it was impossible to tease apart which might be the predisposing factor.

The percent of people infected with multiple pathogens was higher than reported rates from similar studies in Brazil and India [37]–[39]; however, the populations these previous studies sampled tended to be urban and in very different geographical locations than Madagascar. Co-infections in children under five in Madagascar were assessed in a recent report; however, they only documented co-infections between bacteria and parasites or viruses [17]. Given the paucity of data on rates of co-infection with multiple enterobacteria, it is difficult to say whether our data are within the expected range, and certainly this is an area needing further investigation. Furthermore, follow up work should be done to determine if the different co-infection profile of Ank is tied to its more remote location or other variations in behavior or activities from that of the people in Avy or Amb.

We attempted to identify potential risk factors amongst humans for infection with enteric pathogens. People in Amb were significantly more likely to be infected than people in Avy or Ank (Table 4). Water contamination is a notable source of these pathogens [1], [40]. Given the high proportion of open water sources used by the subjects of this study, it was unexpected that there was no significant correlation between fetching water from a closed vs. an open source and their infection status (Table 4). Surprisingly, in the village of Avy, there was a substantial risk associated with people who fetched water from a closed source as opposed to the open sources and ETEC was implicated as the responsible agent (Table 4, 5). These data suggest that one or more of the pumps may be contaminated. It would be insightful to test the pump water directly and determine which families use which pump to see if there is a pattern of bacterial contamination and infection. This finding highlights the importance of these types of studies as follow-up analysis can be focused on areas of interest, such as these pumps.

In addition to the contaminated water source, there was a greater RR for infection with ETEC for people in Avy who reported not boiling their water (Table 5). Interestingly, people that reported always boiling their water before consumption were not at a reduced risk for infection by these bacteria (Table 4). There are several possible explanations. For one, there could be survey bias in that people felt pressure to report that they always boil water when in fact, they do not. This is a risk when having surveys administered in person rather than with complete anonymity. However, given the lower level of literacy and the need for explanation of certain questions, we felt the best way to conduct the surveys was with a native interpreter. Alternatively, coupled with the data from the open versus closed sources of water, this may not be a significant cause of disease transmission overall.

Other factors linked to infection were being under the age of 15 or male. People in Avy who had used medication, either traditional or commercial, in the past four weeks carried a greater risk of infection by ETEC. This finding could be due to several factors. There were a high number of people using either antibiotics (anti-bacterial, -protozoal, and- helmintic) or anti-inflammatories, often times both. It is possible that anti-inflammatory use may hinder the immune response making people susceptible to infection. Moreover, antibiotic misuse may mean a diagnosed infection was not cleared completely. These data could also be indicative of the more worrying trend of antibiotic-resistant bacteria which is well documented in developing nations, due in part to misuse of antibiotics [41], [42]. Further studies are warranted to sequence the strains and whether genes associated with antibiotic resistance are present in these bacteria. Individuals who reported never using toilets had a greater RR of carrying either *V. cholerae* or *Yersinia* spp. Hygiene and sanitation are critical indicators of health [1], [3], [40]. Developing countries tend to have limited sanitation facilities and also have higher rates of infection with various enteric pathogens [40]. While Madagascar has made significant improvements in this area there is still work that needs to be done in providing facilities and changing behavior [15].

Lastly, in Avy, infection with ETEC was significantly associated with having suffered from diarrhea in the past 4 weeks. However, when all villages were factored together, *V. cholerae* was associated with participants having reported diarrhea in the past four weeks (Table 6). Both pathogens are known to cause diarrhea and depending on the study referenced, the location, and the population tested, both have been pointed to as leading causes of infection and disease [4], [17], [39]. Overall, a relatively low association of diarrhea with positive infection status is not surprising. Many of these pathogens can be carried in an asymptomatic state and people often suffer from diarrhea less upon subsequent infections with these enteric pathogens. Asymptomatic carriers can facilitate spread, especially in regions lacking adequate sanitation infrastructure. Meanwhile, repeated infections, especially in children have negative implications on their general health, growth and susceptibility to other infections [43]–[54]. Our study further confirms the role these pathogens play in causing disease in people and are perhaps where future attention should be focused as far as vaccine and treatment efforts.

All livestock species demonstrated relatively low prevalence of all five target pathogens, and there was no correlative risk for people who reported tending livestock vs. those who did not (Table 2 and 4). However, it should be noted that pigs carried a significantly higher risk of harboring one of these bacteria over cattle. This is especially pertinent as pigs have played a key role in other epidemics and pandemics, such as swine flu and Nipah virus [11], [12].

While infection prevalence was relatively low for livestock, rodents had a nearly 2.8 times higher risk of carrying one of the pathogenic intestinal microorganisms over livestock. Moreover, rodents had the second highest overall prevalence at 51% (Table 2). Rodents are common in human and fragmented environments presenting a great opportunity for diseases to move between them, humans, and domestic animals [7], [55]. Peridomestic rodents living in close quarters with human environments are known sources of various diseases including: hantavirus, *Salmonella* spp., *Campylobacter jejuni*, *Giardia* spp. and *Cryptosporidium* spp. [7] [21] However, there was no link between humans having reported touching rodents and an elevated risk of being infected. This could be due to underreporting of contact with rodents or contact people are unaware of, such as while sleeping or rodent fecal matter in their food. Given that *Yersinia pestis* is endemic to Madagascar, exposure to rodents and the fleas they carry is a serious risk for plague in addition to other diseases [25], [26]. Enteric *Yersinia* spp. were detected in humans in all three villages; however, none of the cattle or pigs, the most likely source of enteric *Yersinia,* that we tested were found to carry the bacteria. The lack of positive livestock samples could indicate that there is another animal reservoir for enteric *Yersinia*. Regardless, there were positive samples, indicating that enteric *Yersinia* spp., especially *Y. pseudotuberculosis,* persist in the human population in this region of Madagascar and make follow-up studies to sequence the present strains important.

There are many variables to consider when dissecting the lifecycle and transmission routes of a pathogen. As we expand our understanding of emerging infectious diseases it becomes increasingly clear that the way humans interact with their environment has profound effects on the dispersal of zoonotic pathogens [5], [18], [55]–[59]. This report points to areas for further study, namely water sources and human behavior that may account for the infection status of the human volunteers. Moreover, these data emphasize that humans, livestock, and rodents are all potential sources of pathogenic bacteria and as these groups interact more, the possibility for transmission increases, as does the likelihood of transmission to wildlife such as lemurs [18]. Understanding the host-origin and the subsequent dissemination of a disease at the human-livestock-wildlife interface can aid in combating the spread of these agents. Our work has shed light on the prevalence of various pathogenic bacteria in the human, livestock, and rodent populations in Southeastern Madagascar. More generally, this work has highlighted the complexity of these studies and that generalizations cannot always be drawn even from relatively related populations. What may be a risk factor in one village may not be for another nearby. It is important to take into account the individual as well as the population in studies such as these. Hopefully these findings will help in implementing preventative measures for people, their companion animals and livestock, and peridomestic rodents. More broadly, this work helps to expand our knowledge of disease transmission so that we can better combat these illnesses and enhance the quality of life for these people and others in similar settings.
